# The Sexuality of Adults with Intellectual Disability in Poland

**DOI:** 10.1007/s11195-013-9294-8

**Published:** 2013-03-17

**Authors:** Remigiusz Kijak

**Affiliations:** Department of Special Pedagogy, Pedagogical Faculty, Pedagogical University in Cracow, Ingrarden Street 4, 30-060 Cracow, Poland

**Keywords:** Intellectual disability, Severe and moderate intellectual disability, Sexuality of people with intellectual disability, Sexuality, Sexual experiences, Poland

## Abstract

Sexuality is one of the most important aspects of human life that relates to sex, one's identification, sexual role, sexual preferences, eroticism, pleasure and intimacy. It fulfils such functions as procreative, hedonistic and relationship-building as well as constitutes an integral part of human's personality. The sexuality of people with intellectual disability is a special case - both from medical, pedagogical, psychological and ethical point of view. Little available research shows that it may become a significant factor that modifies their psychological and sexual functioning. The basic poll involved altogether 133 people with mild intellectual disability. The work was carried out in 11 schools and special institutions of three provinces in Poland: kujawsko - pomorskie, wielkopolskie and dolnośląskie (provinces of Kujavy and Pomerania, Great Poland and Lower Silesia) The respondents qualified to take part in the poll constituted a very uniform group - homogenous as regards their age of 18-25 as well as IQ level that was average for the people with higher degree of intellectual disability (HDID). Their age was of importance as in that life period one can observe the formation of first partner relationships with the clear aim of establishing a family. It is accompanied by a quick development of sexual desire and taking up various forms of sexual activity. People with intellectual disability don't form a homogenous group as regards their psychological and sexual development. In this group, one can observe both different forms of clinical mental handicap which definitely affects the whole process of sexual development. The sexual development is delayed by an average period of 3 years. The people with intellectual disability take up mostly autoerotic behaviour whereas partner relationships wthin that group are more seldom. The phenomenon of sexuality of people with higher degree of intellectual disability is an issue that needs further constant analysis. The research has also made it possible to detect what kind of sexual behaviour people with intellectual disability undertake and the value of sexuality for such people. The article deals also with some important dilemmas connected with sexual education and what factors trigger off incorrect sexual reaction including their lack in the above mentioned group.

## Introduction

The sexuality of people with intellectual disability has recently become the topic of professional discussion and public debate. The normalization rule introduced into society made it necessary to understand and accept an intellectually disabled person as a sexual person. Unfortunately there is still a lack of knowledge on people with higher degree of intellectual disability.^1^ As a consequence, this group may struggle with many problems in building satisfactory relationships with a partner and having a successful sexual life. Due to limited research in this field it is difficult to achieve any general conclusion as to the knowledge and sexual activity of people with HDID. The analysis of studies available in literature gives only a general perspective on this issue.

The presented studies are related to three areas: the sexual development of HDID people, knowledge of HDID people on selected aspects of sexual life and selected forms of sexual activities of HDID people. The studies presented in this article have been co-financed by the Polish Ministry of Science and Higher Education under Grant No. N N106 149434.

The studies have been preceded by pilot studies^2^ aimed at determining the scale of the studied problem, proper research procedure as well as verifying adopted research tools. The pilot studies gave several pieces of interesting data: every eighth intellectually disabled woman is unaware of the basic tests she needs to undergo in puberty and every ninth intellectually disabled man does not know how to check his testicles on his own. Every sixth intellectually disabled person fears sexual encounters strongly enough that he/she decides not to start having sex with a partner. Low levels of sexual awareness sometimes become the reason for taking risky sexual behavior. The interest in sexual life among intellectually disabled people is high; however the knowledge on sex, contraception, social and biological sex roles is very low. Every seventh woman and every fifth man showed negative feelings towards masturbation. Sexual activity is low. Intellectually disabled people were unable to clearly determine what the skill to sexually satisfy a partner is. Almost all disabled people in the study declared that they have started sexual activity, admitted that during sex it is important to satisfy a partner’s sexual needs—however they were unable to clearly define that satisfaction.

The present work concentrates on presenting the knowledge of the people questioned on private body parts, fertilization, pregnancy, childbirth and contraception. Various forms of sexual behavior practiced by people with HDID have also been described as well as the evaluation of the sexual development of the group in question.

The work formulates three basic research problems:

1. What is the level of sexual development of the tested people with a higher degree of intellectual disability?
2. What is the level of knowledge of the tested people with a higher degree of intellectual disability on the following subjects:
  - private parts of their bodies?
  - insemination?
  - giving birth?
  - pregnancy?
  - contraception?
3. What forms of sexual activities are taken up by people with a higher degree of intellectual disability?
  - Whether and with what frequency are masturbation activities taken up?
  - Whether and with what frequency are petting activities conducted?
  - Whether and at what age does their sexual life start?

## Method

In the applied research project, the presented material was based on genuine source input—real comments, but described not as individual examples but as a more or less typical common sexual experiences of a group of people with HDID. The input material for such a definition of the issue was provided by the research work with 133 people with a higher degree of ID.

The first method used was an interview with a nurse and a doctor in order to estimate sexual development of participants according to J. M. Tanner’s scale. The next method used was an interview questionnaire (DSMN, [1]).

### Interview Questionnaire

The research work made use, primarily of the technique of a standard interview in the form of a guided conversation with people with HDID. It was prepared according to a precise described sample that comprised a set of questions, their correct sequence as well as the manner of asking them. It was also exceptionally important to provide similar interview circumstances for all the people interviewed. These circumstances were very important for the material collected to be compared and then generalized. As the character of the conducted research was connected with exceptionally sensitive and personal areas, which are usually hidden and not easily shared, it was necessary to put the prepared interview questionnaire and poll to a wide range of consultations. The interview questionnaire was first discussed by psychologists and psychotherapists as well as a psychiatric doctor. Their remarks concerned mainly the applied inventory of notions and consequently their suggestions were contained in the interview questionnaire and elaborated on in such a way as to ensure that they met the criteria of correctness as regards questions asked and applied terminology.

It should be emphasized that the results achieved during the research work with youths with a higher degree of ID were later discussed with their teachers in charge and teachers who had direct contact with them. All suggestions and remarks by these teachers were noted and then taken into account while elaborating the results.

The interview comprised of three stages. Stage 1 was to establish good contact with the person being interviewed. The questions concerned their general physical and mental state (mood) as well as their interests. The aim of that stage was to create an atmosphere of safety and trust. The next stage, called the main one, consisted of conducting the interview according to previously prepared questions. Stage 3—was the stage of ending, summarizing the interview results, explicating potential misunderstandings and a small talk to finish the whole procedure. Each interview was conducted directly with the person with a higher degree of ID and lasted for about 3 or 4 h with breaks.

The research work made use of the following testing tools:

An interview questionnaire from a school nurse destined to evaluate sexual development, an interview questionnaire to evaluate the sexual experiences of the youths with HDID (DSMN). There were two versions of the questionnaire, one for men and one for women.

#### Set of Charts Used During the Interview

The interview questionnaire consists of 3 parts, each of them testing different aspects such as knowledge, behavior and emotions connected with one’s own sexual life. The present work presents only the results obtained from parts I and II of the interview questionnaire i.e. the ones concerning knowledge and behavior. The interview questionnaire had two versions, a version for men and for women and was supplemented by charts that contained notions connected with human sexuality.^3^ The use of visual communication was necessary in many cases. The charts presented objects, activities and features as well as names of presented notions. All the pictures on the charts were schematic and simplified in form. The interview questionnaire questions refer to the description of specific constituting parts of one’s sexual behavior.

- The part concerning knowledge was categorized into 3-scale answers, giving an opportunity to describe the level as low, medium or high. This part dealt with the problems of human sex identification, the physiology of the genitalia of one’s own body and that of the opposite sex, the physiology of fertilization and pregnancy, contraception, sexually transmitted diseases (STD), HIV/AIDS, changes within the body occurring during the adolescent period, body hygiene in that period, the feeling of shame as well as sexual activity in public and private places. In this manuscript there was presented only the level of knowledge on private body parts, fertilization, pregnancy, child delivery and contarception.

##### Knowledge on Private Body Parts

The tested people achieved high scores when they were able to correctly name external attributes of their own and opposite sex (women/men—each 3 attributes). The study subjects were tasked to indicate, in the picture of a man, testicles, penis and pubic hair and in the picture of a woman—breasts, vagina and pubic hair. The score was referred to as a middle when the study subjects named 2 attributes, and too low—when they were unable to indicate any or just one.

##### Knowledge on Insemination

It was found that the tested group could achieve high level of knowledge on physiology of insemination if they could respond to 3 asked questions (whether a woman can become pregnant through kissing, hugging or touching with a man with their clothes on) as well as to explain correctly how the process of insemination takes place. Average results were scored when they could do the same with 1–2 such problems and the tested people had difficulties in explaining the idea of insemination, whereas low score was ascribed when none of the above problems was correctly answered and no explanation of insemination given.

##### Knowledge on Pregnancy

As regards physiology of pregnancy, high score was reserved for the tested people who could determine the time necessary for a full development of an inseminated egg into a human being. Average results were scored by those who, in spite of not giving correct response, were aware that the development of an inseminated egg is a long-lasting process. Low score reflecting very little knowledge on physiology of pregnancy was obtained when the tested people had no idea of pregnancy and could not respond whether it takes long or short time for an inseminated egg to develop into a human being.

##### Knowledge on Giving Birth

In the questions concerning physiology of giving birth, the tested people could score high if they could explain that a woman gives birth to a child through her procreative tracts; average score when they had difficulties in precise explanation of the phenomenon of birth and low score when no explanation was given at all.

##### Knowledge on Contraception

As regards knowledge on contraception, the tested group could score high when named 3 contraceptions methods, average with two and low when no contraception methods were named.

- In the part dealing with undertaking sexual activity, the people interviewed were asked to answer “yes–no” questions, with the possibility to express their opinion in detail. Many of those under study have openly discussed sexual experience. The presented study results are illustrated with several selected sentences in their original wording, expressed by the people under study. The atmosphere of these conversations is difficult to precisely depict, e.g. the voice, the look of eyes, reflection, unsure look, laugh or emotion, the nervous movement of hands, clapping of hands or whispers. A lot of non-verbal information often accompanied words. The expressions have been selected in such a way as to present, as wide as possible, the different opinions of the people under study on the described issue.

### Auxiliary Tools Have Also Been Used in the Studies

#### Nurse (Doctor) Survey on the Sexual Development Assessment of HDID People

The tools were of an auxiliary nature and merely supplemented the conducted studies. The people tested took part in a doctor’s or nurse’s interview that contained questions about the age and time when first menstruation among women and first wet dreams among men appeared. According to these factors, the sexual ages of the people concerned were determined. Every respondent had his/her pubescent stage evaluated applying a simplified J. M. Tanner’s scale.

The level of sexual development and all specific data connected with the somatic aspect of sexual maturity were diagnosed by a school nurse according to the patient’s medical record or by consulting the doctor in charge in the case of incomplete records. The level of development of secondary sexual attributes (features appearing during each stage of adolescence differentiating an adult from a child) such as different sexual tracts and copulative organs, puberty hair (P), hair in the armpit (A), development of genitals (X), breast increase (Y), facial hair were also settled. The test made use of a simplified model of describing sexual development among the tested group and was applied taking account of possibly the most precise and complete interview with a doctor or a nurse. As a result of the above, their sexual development was described according to a modified J. M. Tanner’s scale that finally took the following shape:

1. Pre-pubescent period, (A1, P1, Th1, X1, Y1),
2. Early pubescent period, (A2, P2, Th2, X2, Y2),
3. Middle pubescent period, (A3, P3, Th3, X3, Y3),
4. Late pubescent period, (A4, P4, Th4, X4, Y4),

##### Participants (Information About the Participants of Research)^4^

Bioethical Committee—independent organization giving opinion and supervising clinical trials, established to provide proper protection of human dignity during the studies. The Bioethical Committee controls, among others: the legitimacy, feasiblity and clinical study scheme, anticipated benefit and risks analysis, clinical study protocol correctness. A clinical study cannot start without the consent of a bioethical committee.

The people to be examined were selected according to formal certificates^5^ stating their ID level and the core research work concentrated on people with a higher degree of ID. The basic research work comprised 133 people altogether and was carried out in 11 schools and special institutions in three Polish provinces: Kujawsko—Pomorskie, Wielkopolskie and Dolnośląskie (provinces of Kujavy and Pomerania, Great Poland and Lower Silesia).

The respondents qualified to take part in the research constituted a uniform group—homogenous as regards their age of 18–25 as well as an IQ level that was average for the people with a higher degree of ID. Their age was of importance, as in that life period one can observe the creation of first partner relationships with the aim of establishing a family.^6^ It is accompanied by the quick development of sexual desire and the beginning of various forms of sexual contact (Tables 1, 2).

**Table 1:** Gender of the tested people

Women		Men		Total
N	%	N	%	
42	32	91	68	133

*Source* the author’s own research

**Table 2:** Age of the tested people

17–21		22–25		Total
N	%	N	%	
96	72	37	28	133

*Source* the author’s own research

## Results

### Sexual Development

In the tested group, most people are placed in the middle (40 %) or late (49 %) stage of pubescence with only 10 % at an early pubescent stage, whereas with one person a pre-pubescent period was diagnosed. A breakdown of the factor within that group is presented in the Table 3.

**Table 3:** The evaluation of the sexual development of the tested group

Pre-pubescent period		Early pubescence		Middle pubescence		Late pubescence		Total
N	%	N	%	N	%	N	%	
1	1	13	10	53	40	66	49	133

*Source* the author’s own research

The tests tried to determine the age when the first wet dreams and menstruation took place. The presented data revealed that people with a higher degree of ID are not a homogenous group and the occurrence of the first menstruation and wet dreams may take place even 3 years later than among fit people.

On the basis of the research, one may conclude that the first wet dreams among men with a higher degree of ID took place at the age of 14.5, whereas in the case of women—first menstruation at 13.4 years of age. There are different opinions as to the age when fully fit women and men start to sexually mature. It can be stated that although the process is individually differentiated, generally women tend to sexually mature faster than men. On average, women start to mature sexually at the age of 10.6–13 years whereas men—12.6–15 years (cf. [2], p. 310). Kucz ([3], pp. 35–59) indicates that this age among women is between 8 and 14 years and in the case of men between 10 and 16 years (Table 4).

**Table 4:** The evaluation of the sexual development of the tested group

Women with ID		Men with ID		General index of men and women with ID	
N	Average age of first menstruation	N	Average age of first wet dreams	N	Average age of first symptoms of sexual maturity (menstruation and wet dreams) in the group of men and women with ID
42	13.4	92	14.5	133	14.1

*Source* the author’s own research

### Sexual Activity

#### Masturbation

In the study group we “have managed” to determine that 101 people masturbated, i.e. 76 % of the study group. I am writing “we have managed” here as there were favorable study circumstances, i.e. an atmosphere of full respect and acceptance which made it possible to talk about the issue without any embarrassment or shame and without breaching the subjects’ right to privacy and intimacy.

The group of men with HDID masturbated almost twice as often (89 %) as women (48 %). As a result of the analysis, one can state that autoerotism is the most frequent form of their sexual behavior. The analysis of masturbation frequency showed that such an activity was carried out mostly a few times a week (41 %); whereas as many as 18 % of the tested youths masturbated a few times a month, 28 % once a day and 14 % a few times a day.

Masturbation can be stated to be a normal sexual practice when it is undertaken at the age excluding partner relations. The masturbation acceptance is of a conditional nature, depending on age, duration and circumstances of the person masturbating. Based on the studies conducted it can be stated that for the majority of the study subjects masturbation has a developmental nature and constitutes an integral and natural phase of sexual development. However the interpretation of the study results requires reference to certain theoretical positions which, among others, perceive masturbation as the only form of sexual activity accessible for the disabled. It is difficult to explicitly agree with this thesis. As a result of studies, it has been discovered that the majority of the studied intellectually disabled teenagers, who masturbated, have not excluded taking up sexual activity with a partner (Table 5).

**Table 5:** Frequency of masturbation

	A few times a day		Once a day		A few times a week		A few times a month		A few times a year		More seldom	
	N	%	N	%	N	%	N	%	N	%	N	%
Sample group	14	14	28	28	41	41	18	18	0	0	0	0

*Source* the author’s own research

Looking further into masturbation, we can go through the places where the intellectually disabled teenagers studied, masturbated. 100 % of the teenagers studied, masturbated at home on their own. The exact analysis of empirical material allowed drawing extra conclusions. It has been determined that the studied intellectually disabled teenagers masturbated in other places as well, outside home. These places included schools, parks, squares, public toilets, shops, trams, buses, and forests as well as healthcare facilities (Table 6).

**Table 6:** Masturbation (study group N = 101, control group N = 89) Place of masturbation

	At home, on their own		At home, in the presence of others		At school, on their own		At school, in the presence of others		In public places	
	N	%	N	%	N	%	N	%	N	%
Study group	101	100	21	21	23	23	19	19	10	10
Control group	89	100	0	0	0	0	0	0	0	0

*Source* author’s own study

During the studies it has also been determined that 7 % of the studied teenagers stimulate themselves in an untypical manner. The teenagers studied admitted to masturbating with tools, certain objects or to masturbating in a way other than a natural one. The study subjects masturbate using grease, food, furniture and even vacuum cleaners. Such masturbation can be determined as dangerous, mainly due to the fact that it fixes a certain, repeatable chain of strange rituals, often impossible to use in a partner relationship, and may result in a pleasure decrease.

#### Petting and First Sexual Intercourse

People with HDID do take up sexual activities and, similarly to the group of those intellectually fit, one can observe differences as regards this aspect. The evaluation of sexual behavior showed that 13 % of people with a higher degree of ID had already started some kind of sexual activity, and the average age of doing this does not differ from that of intellectually fit people and amounts to 18 years of age. The age of first sexual intercourse among men averaged 17.5 years of age, whereas for women, 19 years of age.

Although women and men with HDID have their first sexual intercourse later than intellectually fit young people (the difference amounts to 3 years) it is not justifiable, however, to say this is based only on the collected results that we deal with here with regards to the retardation and acceleration of that activity in both groups respectively. On the other hand, such interpretation cannot be completely excluded. All discussions concerning the topic of the optimal time of first sexual intercourse within the group of people with HDID reveal ambiguous opinions, mainly because taking up sexual activities within that group is an exceptionally rare phenomenon. The main stimuli of taking such decision are: curiosity (39 %), need to prove one’s adulthood (33 %), emotional relation with one’s partner (22 %) as well as encouragement from others (17 %) (Tables 7, 8).

**Table 7:** Age of the first sexual contacts

	At 15 years of age		16–18 years		18–20 years		Over 20 years of age	
	N	%	N	%	N	%	N	%
Sample group	0	0	2	11	3	17	13	72

*Source* the author’s own research

**Table 8:** Reasons of starting sexual contacts

	Seduction		Curiosity		Need to feel like an adult		Emotional relations		Feeling of boredom	
	N	%	N	%	N	%	N	%	N	%
Sample group	3	17	7	39	6	33	4	22	0	0

*Source* the author’s own research

The intellectually disabled teenagers studied, usually chose persons of similar age for their sexual partner. In all the cases studied, the sexual partner was also an intellectually disabled person (Table 9).

**Table 9:** Sexual initiation (study group N = 18, age of sexual partner)

Under 15 years		Peer		Much older	
N	%	N	%	N	%
0	0	18	100	0	0

*Source* the author’s own research

In three cases a sexual partner was a person of the same sex. In the remaining cases a sexual partner was of the opposite sex (Table 10).

**Table 10:** Sexual initiation (study group N = 18) Sex of sexual partner

The same sex		The opposite sex	
N	%	N	%
3	16	15	83

*Source* the author’s own research

Based on research, one can assume that for the majority of the people tested with HDID, petting involved the caressing of their partners’ whole body but without sexual intercourse. Most of them started that sexual activity out of curiosity, which accounted for 85 % of cases. As many as 35 % of them also considered emotional relations—understood a need to get closer to another person—as an important factor. The frequency of petting depended on having a stable partner. Most people questioned who admitted to petting said that they had done it only once; however, they added that if they had been able to repeat that experience, they would have done so. Only 11 % admitted regular petting with their partner, all of whom had a stable partner. For the people questioned the surrounding atmosphere also mattered; in most cases, that happened at home while their parents or other members of their families were absent. Each person questioned said that it was a very important experience, which could help him/her get closer to their partners. Also the differences between men and women’s behavior while petting were noted. The men mainly initiated that kind of activity, while the women acted rather passively. It is also worth noting that for men petting was the preliminary stage to having full intercourse, while women considered that activity as sufficient to satisfy them. We can assume that for the youths tested, petting was an important element on their way to full sexual expression (Table 11).

**Table 11:** Petting

	People with ID of the same sex		People with ID of the opposite sex		Fit people of the same sex		Fit people of the opposite sex		Related partner	
	N	%	N	%	N	%	N	%	N	%
Sample group	3	9	30	94	1	3	1	3	0	0

*Source* the author’s own research

To illustrate the presented analysis one may quote a few chosen comments from the tested participants:

When Ann and I touched each other on the bottom—that was a cool feeling. I was seventeen when I had sex with a girl for the first time. She was the same age as me. I did it out of curiosity. She was very scared and even cried. I thought that something bad had happened to her. It was not too good. But now, we have sex and it is really cool (man, aged 19),

I have already had sex. The girl lived in my district. She doesn’t study here anymore as she has already completed school. She left school last year. Now she spends time at home. I did it out of curiosity to find out how what it is like; one has to do it eventually. The girl cried afterwards and she said that it hurt her or something like that. It didn’t hurt me. It was not bad but now it has got better. Some of my friends have already had sex. I also wanted to do it. Now, I have a different girlfriend. I have also had sex with her a few times. I am a little worried about getting her pregnant so I don’t do it very often. (man, aged 21).

The conducted empirical analysis shows that 15 people took up sexual activity after the first sexual encounter, which accounted for 11 % of the total participants. 10 people continued having sex with the same partner and 8 had more than one sexual partner (in most cases the number varied from one to three). In one case, a person had homosexual relations with a partner whom he/she considered his/her friend. These people studied in the same school and lived in a dormitory. They had sexual contact mostly at weekends when they were alone in the dormitory.

It was found that only 39 % of the tested people used any contraceptives. The rest did not use any form of protection against pregnancy or sexually transmitted diseases. The main reasons for not doing so, were a lack of knowledge and a feeling of ‘discomfort ‘or a feeling of disgust as well as difficulty in applying the contraception (Table 12).

**Table 12:** Contraception

	Use		Do not use		Total
	N	%	N	%	
Sample group	7	39	11	61	18

*Source* the author’s own research

### Knowledge of Sexuality

#### Private Body Parts

The conducted research allows me to claim that as many as 90 % of the youths examined with a higher degree of ID can differentiate physiological attribute peculiar to their own sex. This skill as regards the opposite sex was displayed by 10 % fewer respondents. 7 % of the youths tested had difficulty in telling apart one’s own private body parts, with 16 % in the case of the opposite sex. Only 4 % could not identify correctly one’s own private body parts and 7 % could not do it with the opposite sex (Table 13).

**Table 13:** Knowledge of one’s own private body parts and those of the opposite sex

Physiology of one’s own private body parts	High level		Medium level		Low level		Total
	N	%	N	%	N	%	
Knowing one’s own private body parts	119	89	9	7	5	4	133
Knowing private body parts of the opposite sex	102	77	22	16	9	7	133

*Source* the author’s own research

#### Fertilization

Following the analysis, it was found that 52 % of the people tested with a higher degree of ID could correctly describe the idea of fertilization. They knew how a child is conceived. They gave incorrect answers when asked if a woman could become pregnant through kissing a man, hugging or touching through clothing. An average level of knowledge was scored by 11 % but as many as 37 % achieved a low score. The youths lacking knowledge on fertilization believed that a man does not take part in the process of fertilization. It was believed that a man’s responsibility consisted of supporting a family by taking up work (Table 14).

**Table 14:** Knowledge about fertilization

Physiology of fertilization, pregnancy, childbirth and contraception		High level		Medium level		Low level		Total
		N	%	N	%	N	%	
Fertlization	Sample group	69	52	15	11	49	37	133

*Source* the author’s own research

#### Pregnancy

The conducted research revealed a severe lack of knowledge on pregnancy. The sample group could not explain the idea of pregnancy. Almost 65 % did not know how long it takes for a fertilized egg to develop into an independent human being and they could not describe if the time was long or short. Their answers concerning that topic were simplified and often not correct. 14 % of the youths with a higher degree of ID showed average (medium level) knowledge on the physiology of pregnancy. They knew that a child is conceived within a female’s body, develops there and after achieving final form is delivered. In spite of giving imprecise and often wrong answers, they were aware that the development of a fertilized egg into a human being is a long process but only 21 % of them could precisely describe the time necessary for it (Table 15).

**Table 15:** Knowledge of the physiology of pregnancy

Physiology of fertilization, pregnancy, childbirth and contraception		High level		Medium level		Low level		Total
		N	%	N	%	N	%	
Pregnancy	Sample group	28	21	19	14	86	65	133

*Source* the author’s own research

#### Childbirth

As many as 81 % of the youths with a higher degree of ID knew very little about childbirth. When asked, they gave made up answers on the topic. The most frequent answer concerned women staying in hospital. Very few of them made use of proper terminology such as ‘giving birth’ to a child. Most respondents associated childbirth with something difficult and painful. They believed that a woman gives birth to a child on a table in hospital in the presence of a doctor but at this point their knowledge ended.

To illustrate the above, one may quote a few chosen comments made by the sample group of people with HDID.

The woman gets pregnant and feeds her baby with her breast. To have a baby, a woman must have sex with a man. Then, the woman and man have an orgasm and the man gets her pregnant. The man has sperm and it gets into the womb so a fetus is created. A child is in the stomach and when it grows, the woman will have a baby. When the woman’s stomach is growing, she goes to a doctor. She should buy a pregnancy test; she may feel sick. The doctor touches her stomach and the fetus can be seen on a computer screen. I have seen it on TV on a program. Men can’t be pregnant and give birth to a child. The woman gives birth by pushing to get the child out. When she can’t do it, they cut her stomach and get the child out (man, aged 20).

#### Contraception

Another comment proves that the youth in question had some knowledge on fertilization, pregnancy and childbirth but even then, it is incomplete and not precise enough. The people questioned knew very little about childbirth. Very often, one can find in their opinions such expressions as ‘a child is born through a womb, navel or breast in hospital.’

It was determined that only 10 % of the tested youths understood the notion of contraception. These people were able to name at least three methods of contraception; condoms and contraceptive pills were mentioned most often.

## Conclusion

The obtained results show that adults with a higher degree of ID have certain sexual experience but not enough knowledge on human sexual life. According to the conducted research, it was found that the group in question mostly identify with their own sex and can differentiate their gender from the opposite one. In most cases, the youths surveyed could match the external attributes to the proper sex and they seem to accept the physical aspect of their gender.

Masturbation and petting were found to be the most frequent ways of expressing one’s sexual needs, with masturbation playing the major role, whereas touching and sexual intercourse proved less popular. The masturbation activities were found to occur in various circumstances, most frequently in intimate situations. A deficiency of information on how to deal properly with this form of sexual expression results in the fact that people with HDID often make use of pathological forms of masturbation jeopardizing their health and even life. It has also been found that the youths surveyed prefer autoerotic to socially-oriented sexual activities (the empirical analysis showed that 76 % of the youths examined with a higher degree of ID masturbated) Socially-oriented sexual activities such as kissing, petting and sexual intercourse were found to be rather rare. As many as 24 % had petting experiences, while only 14 % started having sexual intercourse. Men, more often than women, were involved in socially-oriented sexual activities.

The phenomenon of the sexuality of people with a higher degree of ID is an issue that still needs constant analysis. It is mainly connected with the change of attitude towards perceiving and understanding disability. One may claim that we are undergoing a process of shifting from taking a strictly biological perspective towards sexual rehabilitation. Among other factors, the time of making that shift is connected with granting certain rights and sexual freedom to those people. However, there is a lot of evidence to suggest that we still deal with the problem by infantilizing people with HDID.

People with HDID masturbate in various circumstances; in most cases, however, it takes place at home. The higher the degree of ID, the more often it happens that masturbation may take place in the presence of family members, during school classes or somewhere in the street. It should be mentioned that among these people, masturbation appears not only as a response to lowered intellectual abilities but more often is a result of improper socialization and a lack of school education. It may also be a form of satisfying one’s emotional hunger, especially, in the case of people who experience some rejection, lack of acceptance, love and caressing or live in loneliness and isolation and are not able to verbalize their emotional state of mind. According to the research work conducted in the 1970′s, c. by Edmonson and Wish ([4], p. 11–18) concerning the sexual experiences of men with ID, one third of such people admitted that masturbation and heterosexual intercourse were not proper activities whereas a large part described homosexuality as a bad activity. These results show that the majority of these men maintain a negative attitude towards expressing one’s sexuality. In opposition to the above research, Timmers et al.([5], p. 27–39) pointed out that a large percentage of people with HDID had positive attitudes towards their masturbation and masturbation in general. In some more recent research, it was found that a higher percentage of people with HDID had a positive attitude towards some aspects of sexuality but in some areas their negative attitudes prevailed. McCabe and Cummins ([6], pp. 13–22) found that the majority of participants of their research had positive attitudes towards sexual intercourse, but only half had positive attitudes towards masturbation, oral sex and homosexuality.

The research results by Goldstein [7] conducted on a small group of men and women with ID, aged 16–28, showed that only 15 % of the people from the group examined had experienced their first sexual intercourse and the average age of having it was 24 years of age. The emotional reactions were rather negative and the behavior was accompanied by such feelings as shame, a sense of sin and disappointment. In the opinion of almost all the people questioned, their first sexual intercourse unfavorably influenced their perception of sexuality. The frequency of sexual contact for 16 % of men and 5 % of women was insufficient to such an extent that they felt that this aspect of their lives remained unfulfilled.

There is a constant need for studies in this field. The presented results are not extensive and constitute merely an invitation to discuss the sexuality of HDID people.

## Footnotes

1 In Polish we use the term “higher degree of intellectual disability” for moderate and severe intellectual disability.

2 The study group consisted of 40 people with HDID: 20 women and 20 men with HDID from Poland.

3 As the literature does not offer any ready visual aids (pictures) to describe human sexuality, the author had to create ones for the sake of the research work.

4 The consents for participation in research had been obtained from participants, their parents and principals of schools. The studies were co-financed by the Polish Ministry of Science and Higher Education thanks to a research grant. Therefore apart from the consents of the parents of the study subjects and the headmasters of the institutions, additionally a statement on the inviolability of freedom and personal rights of study subjects was submitted to the Polish Bioethical Committee. The study subjects were not legally incapacitated.

5 Formal certificates means disability diagnosis. In Poland disability is diagnozed in psychological and pedagogical centres with relevant background using examinations and tests.

6 According to Hevighurst’s Model.
